# A Novel Biological Index for Predicting Neoadjuvant Treatment Response in HER2-Positive Breast Cancer: The Tumor-Immune-Proliferation-Inflammation (TIPI) Score

**DOI:** 10.3390/jcm15083118

**Published:** 2026-04-19

**Authors:** Erdem Sünger, Harun Muğlu, Mehmet Haluk Yücel, Ebru Engin Delipoyraz, Maral Martin Mıldanoğlu, Hakan Özçelik, Sena Fidan, Cihat Terzioğlu, Burçin Çakan Demirel, Jamshid Hamdard, Yasin Kutlu, Özgür Açıkgöz, Aslı Çakır, Mesut Şeker, Ahmet Bilici

**Affiliations:** 1 Department of Medical Oncology, Faculty of Medicine, Istanbul Medipol University, Istanbul 34214, Türkiye; mhalukyucel@gmail.com (M.H.Y.); drebruengin@gmail.com (E.E.D.); mmildanoglu@gmail.com (M.M.M.); hknozcelikk@gmail.com (H.Ö.); senafidan03@gmail.com (S.F.); cihatterzioglu@gmail.com (C.T.); burcin.cakandemirel@gmail.com (B.Ç.D.); jamshidhamdard@hotmail.com (J.H.); dryasinkutlu@gmail.com (Y.K.); ozgur_acikgoz@yahoo.com (Ö.A.); drmesutseker@gmail.com (M.Ş.); ahmetknower@yahoo.com (A.B.); 2 Department of Medical Oncology, Bagcilar Training and Research Hospital, University of Health Sciences, Istanbul 34214, Türkiye; hm1635@hotmail.com; 3 Department of Medical Pathology, Faculty of Medicine, Istanbul Medipol University, Istanbul 34214, Türkiye; acakir@medipol.edu.tr

**Keywords:** HER2-positive breast cancer, neoadjuvant therapy, pathological complete response (pCR), TIPI score, tumor-infiltrating lymphocytes (TILs)

## Abstract

**Objective:** To evaluate the Tumor-Immune-Proliferation-Inflammation (TIPI) score as a composite biomarker for predicting pathological complete response (pCR) in human epidermal growth factor receptor 2 (HER2)-positive breast cancer treated with neoadjuvant therapy. **Methods:** This retrospective single-center study included 75 patients with HER2-positive invasive breast cancer treated with neoadjuvant chemotherapy plus dual anti-HER2 blockade (trastuzumab and pertuzumab). The association between the TIPI score and pCR was assessed using receiver operating characteristic (ROC) analysis and logistic regression. **Results:** pCR was achieved in 34 patients (45.3%). The optimal TIPI cut-off was 11.41. Patients with high TIPI scores had a higher pCR rate than those with low TIPI scores (56.3% vs. 25.9%, *p* = 0.016). However, the discriminative performance of the score was modest (AUC 0.598, 95% CI: 0.467–0.730; *p* = 0.145). In the adjusted analysis, hormone receptor negativity remained the most consistent factor associated with pCR. **Conclusions:** The TIPI score was developed as a preliminary composite model integrating selected tumor- and host-related biological variables and showed an exploratory association with pCR in this single-center HER2-positive cohort. Given the modest discriminative performance and lack of external validation, these findings should be interpreted cautiously. Further validation in larger independent cohorts is required before the score can be considered for clinical stratification or implementation.

## 1. Introduction

Human epidermal growth factor receptor 2 (HER2)-positive breast cancer represents approximately 15–20% of all breast cancer cases and is a molecular subtype historically characterized by its aggressive clinical course. Currently, combination regimens consisting of neoadjuvant chemotherapy (NACT) and anti-HER2 blockade have become the standard of care for this patient population, yielding high rates of pathological complete response (pCR). Achievement of pCR following neoadjuvant therapy not only reflects an excellent response to treatment but is also recognized as an important surrogate endpoint for long-term event-free survival (EFS) and overall survival (OS) [1]. Nevertheless, in patients who do not achieve pCR, the extent of residual disease burden remains a critical factor influencing prognosis [2,3].

Conventional clinicopathological parameters, such as tumor size and nodal status, may be insufficient to fully capture the complex biology underlying heterogeneous treatment responses and the host immune response. Indeed, contemporary data have shown that even advanced radiomic imaging methods do not significantly improve pCR prediction beyond standard clinicopathological variables [4]. These limitations highlight the need for biologically based, accessible, and cost-effective predictive indices.

The Ki-67 index, which reflects the proliferative capacity of the tumor, is directly associated with chemosensitivity and is a widely used marker for predicting pCR [5,6]. Similarly, the predictive and prognostic value of stromal tumor-infiltrating lymphocytes (TILs), which reflect immune activity within the tumor microenvironment, has been validated in numerous pivotal studies, particularly in HER2-positive breast cancer [7,8,9,10]. Standardized TIL assessment, as established by international working group recommendations, highlights the critical role of the immune microenvironment in the success of neoadjuvant therapy [11,12,13]. Contemporary literature also indicates that high baseline TIL levels may enhance the efficacy of anti-HER2 therapies through antibody-dependent cellular cytotoxicity (ADCC) and may improve long-term survival outcomes [14,15].

In addition to the local tumor microenvironment, the antitumor response is also influenced by systemic inflammatory status. Hematological indices such as the Systemic Immune-Inflammation Index (SII) and the Pan-Immune-Inflammation Value (PIV), which integrate neutrophil, lymphocyte, and platelet counts, have emerged as relevant systemic biomarkers associated with treatment resistance and poorer prognosis in HER2-positive breast cancer [16,17,18]. A higher systemic inflammatory burden has been associated with lower pCR rates, likely through suppression of effective antitumor immune responses [19,20,21].

Although several variables have been associated with response to neoadjuvant therapy in HER2-positive breast cancer, most currently available approaches remain fragmented. Conventional clinicopathological variables provide limited biological resolution, while individual biomarkers such as Ki-67, stromal TILs, histological grade, or systemic inflammatory indices are typically evaluated in isolation. At the other end of the spectrum, genomic assays may offer more refined prediction but are not universally accessible in routine practice. Accordingly, a practical gap remains for a pretreatment composite model that integrates tumor-intrinsic aggressiveness, immune microenvironment, and systemic inflammatory status using routinely available data. Recent studies in HER2-positive breast cancer have further supported the relevance of pretreatment systemic immune-inflammatory indicators in the neoadjuvant setting, showing that inflammatory blood markers and systemic inflammation indices may contribute to pCR stratification and complement routine clinicopathological assessment [22,23].

The aim of this study was to evaluate the predictive value of the Tumor-Immune-Proliferation-Inflammation (TIPI) score for pathological complete response in patients with HER2-positive breast cancer receiving neoadjuvant therapy.

## 2. Materials and Methods

### 2.1. Study Design and Ethical Approval

This study was designed as a single-center, retrospective observational cohort study. The study protocol was approved by the Non-Interventional Clinical Research Ethics Committee of Istanbul Medipol University (approval date: 11 December 2025; decision no: 1504). All procedures were conducted in accordance with the Declaration of Helsinki, and all patient data were anonymized prior to analysis to ensure confidentiality.

### 2.2. Patient Selection and Cohort Characteristics

A total of 75 female patients with HER2-positive invasive breast cancer who received neoadjuvant chemotherapy in combination with dual anti-HER2 blockade (trastuzumab plus pertuzumab) at the Department of Medical Oncology, Istanbul Medipol University Hospital, were enrolled in this study. The inclusion criteria were as follows: age ≥18 years; availability of complete pretreatment core needle biopsy pathology reports providing the Ki-67 proliferation index, stromal TIL percentage, and histological grade; and availability of baseline complete blood count parameters for calculation of the SII. Exclusion criteria were metastatic disease, discontinuation of neoadjuvant treatment, missing pathological or laboratory records, bilateral tumors, and male sex.

### 2.3. Pathological Assessment and Biomarkers

The primary endpoint of the study was pCR, defined as the absence of invasive tumor cells in both the surgical specimen and the excised lymph nodes (ypT0/is ypN0) [1,24,25]. Residual disease burden was evaluated using the Residual Cancer Burden (RCB) classification system [2,3]. HER2 status was assessed on pretreatment core needle biopsy specimens using immunohistochemistry (IHC), with reflex in situ hybridization (ISH) testing for equivocal (IHC 2+) cases, according to American Society of Clinical Oncology/College of American Pathologists (ASCO/CAP) recommendations in effect at the time of diagnosis [26]. HER2-positive disease was defined as IHC 3+ staining or IHC 2+ with ISH-confirmed amplification. Only patients with confirmed HER2-positive tumors were included in the study. TILs percentages were scored as the proportion of the stromal area occupied by mononuclear cells, following the standardized methodology recommended by the International TILs Working Group [11,12].

**Figure 1:**
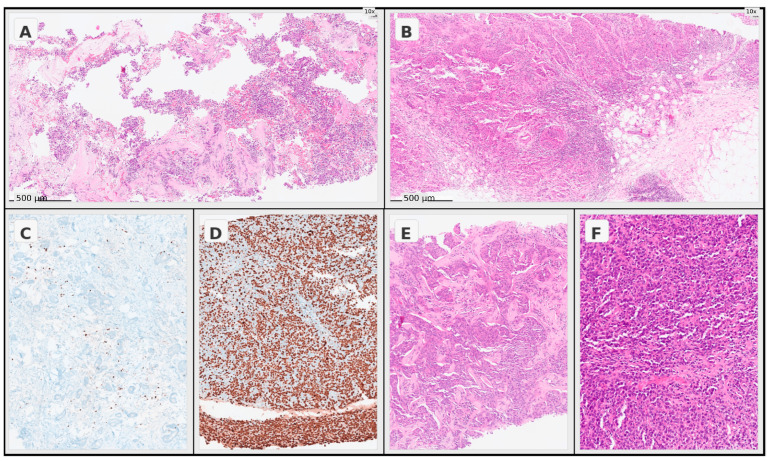
Representative pathological images illustrating the practical assessment of TIPI score components in pretreatment core biopsy specimens. (**A**) Low stromal TILs on hematoxylin-eosin (H&E) staining (10×). (**B**) High stromal TILs on H&E staining (10×). (**C**) Lower Ki-67 immunohistochemical expression (10×). (**D**) Higher Ki-67 immunohistochemical expression (10×). (**E**) Representative invasive carcinoma morphology consistent with histological grade 2 on H&E staining (10×). (**F**) Representative invasive carcinoma morphology consistent with histological grade 3 on H&E staining (20×).

Ki-67 labeling index data were extracted from pretreatment core needle biopsy pathology reports and recorded as continuous percentage values. Ki-67 was used as a continuous variable in the TIPI formula and in the primary continuous-variable analyses. For descriptive baseline comparison only, Ki-67 was additionally categorized using a threshold of 20% (≤20% vs. >20%), as presented in Table 1. Ki-67 and histological grade were assessed as part of routine institutional breast pathology evaluation on pretreatment core needle biopsy specimens. Histological grading was performed according to the Nottingham histologic grading system, as documented in the original pathology reports [27]. No formal central pathology re-review or interobserver variability analysis was performed in this retrospective cohort. Representative pretreatment core biopsy images illustrating stromal TIL assessment, Ki-67 immunohistochemical staining, and histological grade evaluation are shown in Figure 1.

### 2.4. TIPI Score Formulation and Inflammation Index

The SII was calculated as (platelet count × neutrophil count)/lymphocyte count [16,28]. Neutrophil, lymphocyte, and platelet counts were obtained from routine baseline complete blood count testing performed before initiation of neoadjuvant therapy. Laboratory values were retrieved retrospectively from the institutional electronic medical records and hospital laboratory system. The TIPI score was then calculated using the following formula: $TIPI=\frac{Ki-67\left(\%\right)+stromal\;TILs\;(\%)+(10\times Histological\;Grade)}{1+\frac{SII}{1000}}$.

The weighting scheme was intentionally designed as a biologically informed and clinically interpretable heuristic model rather than a data-driven optimized algorithm. Because Ki-67 and stromal TILs are expressed as percentage values, whereas histological grade is an ordinal variable, grade was multiplied by 10 to place it on a more comparable numeric scale within the numerator and to preserve its contribution as a marker of morphological aggressiveness. Likewise, SII was divided by 1000 to reduce numerical dominance of the denominator and to improve interpretability of the composite score. This denominator structure was also intended to reflect the immunosuppressive influence of systemic inflammatory burden on antitumor response [19,20].

### 2.5. Statistical Analysis

Statistical analyses were performed using IBM SPSS Statistics software, version 27.0 (IBM Corp., Armonk, NY, USA). The distribution of continuous variables was assessed using the Shapiro–Wilk test. Associations between pCR and clinicopathological variables were analyzed using the chi-square test or Fisher’s exact test, as appropriate. The predictive performance of the TIPI score for pCR was evaluated using receiver operating characteristic (ROC) curve analysis, and the optimal cut-off value was determined by the Youden index. Univariate and multivariable logistic regression models were constructed to identify independent predictors of pCR. Variables considered clinically relevant and/or significant in univariable analysis were entered into the multivariable logistic regression model using the Enter method, including TIPI risk group, HR status, histological grade, and clinical stage. Model fit was assessed using the Omnibus test of model coefficients (*p* = 0.025) and the Nagelkerke R^2^ (0.256). Odds ratios (ORs) and 95% confidence intervals (CIs) were calculated to evaluate the strength of associations. For all analyses, a *p*-value of <0.05 was considered statistically significant.

## 3. Results

### 3.1. Demographic and Baseline Clinicopathological Characteristics

Baseline demographic, clinical, and laboratory characteristics of the 75 patients with HER2-positive breast cancer are summarized in Table 1. The median age was 45 years (range, 24–78), and 58.7% (n = 44) of the cohort was premenopausal. The mean baseline tumor size was 28.8 ± 15.7 mm. In terms of clinical stage distribution, 50.7% of patients had stage IIA disease. Following NACT, pCR was achieved in 45.3% (n = 34) of the cohort.

### 3.2. Analysis of Continuous Variables and TIPI Score Performance

When evaluated as a continuous variable, patients who achieved pCR exhibited significantly higher stromal TIL levels than those who did not (26.5% vs. 13.4%; *p* = 0.017). Although the Ki-67 percentage was higher in the pCR group, the difference did not reach statistical significance (52.5% vs. 45.1%; *p* = 0.136).

**Figure 2:**
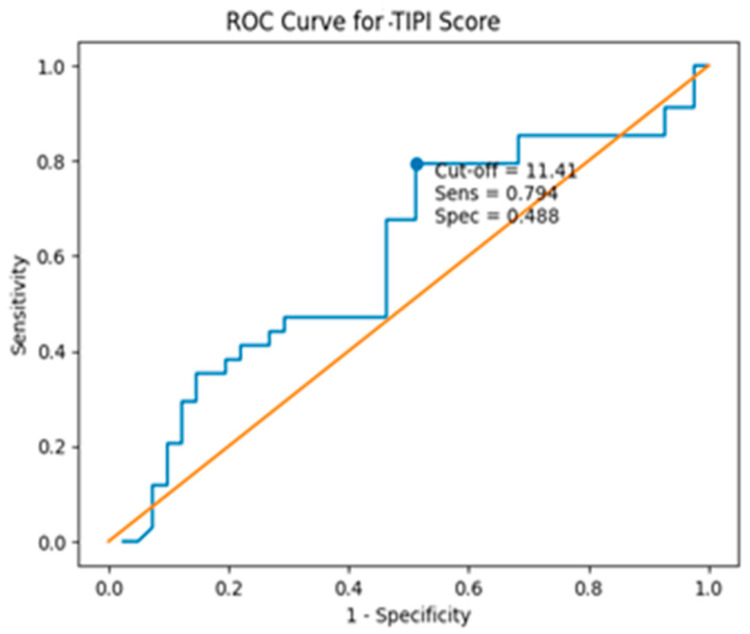
ROC curve analysis of the TIPI score.

The discriminative performance of the TIPI score was modest area under the curve (AUC) 0.598; 95% CI: 0.467–0.730; *p* = 0.145. The optimal cut-off value identified by the Youden index was 11.41, corresponding to a sensitivity of 79.4% and a specificity of 48.8% (Figure 2).

**Figure 3:**
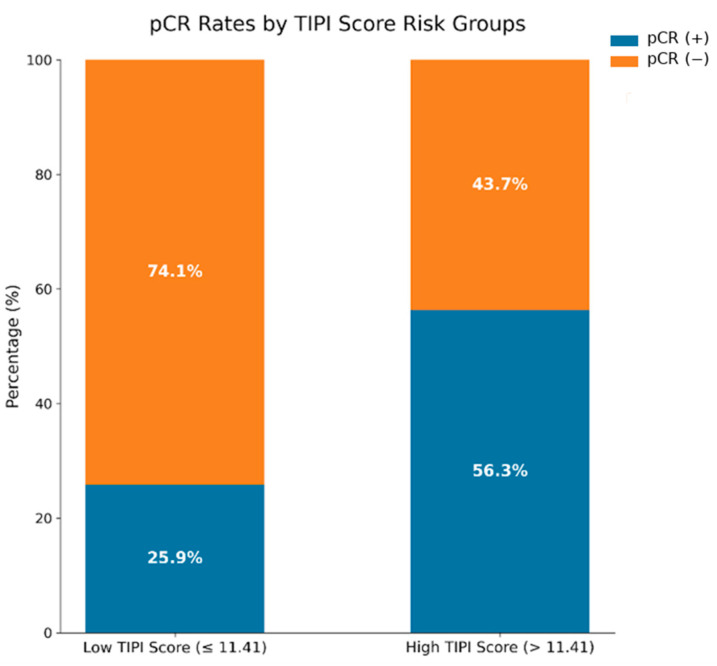
Comparison of pCR rates across TIPI risk groups.

Using this cut-off value, the pCR rate was 56.3% (27/48) in the High TIPI (>11.41) group, compared with 25.9% (7/27) in the Low TIPI (≤11.41) group. This difference in pCR rates between the TIPI risk groups was statistically significant (*p* = 0.016) (Figure 3).

### 3.3. Multivariable Analysis Results

In the multivariable model, HR negativity remained the most consistent factor associated with pCR (OR: 3.765, 95% CI: 1.252–11.321; *p* = 0.018). Although patients in the High TIPI group showed higher adjusted odds of pCR than those in the Low TIPI group, the wide confidence interval indicates limited precision and the result should therefore be interpreted cautiously (OR: 2.427, 95% CI: 0.791–7.462; *p* = 0.121). Similarly, histological grade (G3) did not retain independent significance after adjustment (OR: 2.825, 95% CI: 0.912–8.772; *p* = 0.072) (Table 2).

## 4. Discussion

The achievement of pCR following NACT is an important surrogate outcome measure in HER2-positive breast cancer [1]. However, the limited ability of individual parameters to account for heterogeneous treatment responses has intensified the need for more holistic and biologically driven models. In this context, the TIPI score was designed to integrate local tumor aggressiveness (Ki-67 and histological grade), the immune microenvironment (TILs), and systemic inflammatory burden (SII) into a unified mathematical framework.

A distinctive feature of the TIPI score is its normalization of both promoting and inhibitory biological factors. Ki-67 and TILs, located in the numerator of the formula, are among the parameters most consistently associated with pCR in the literature [5,7]. Whereas elevated Ki-67 levels reflect the intrinsic chemosensitivity of the tumor, increased stromal TILs indicate the strength of the host antitumor immune response [8,9]. In our cohort, higher TIPI scores were associated with higher pCR rates after dichotomization. However, given the modest discriminative performance of the score and the limited precision of the multivariable estimates, these findings should be interpreted cautiously and viewed primarily as supportive of biological risk stratification rather than definitive standalone prediction.

Previous studies examining the relationship between SII and pCR or prognosis in HER2-positive breast cancer have highlighted the importance of systemic inflammatory burden [16,28]. However, these parameters have generally been evaluated independently of local tumor biology. In our study, placing SII in the denominator was intended to reflect the immunosuppressive role of high systemic inflammation, characterized by lymphopenia accompanied by neutrophilia and thrombocytosis [19,20]. This distinction helps explain why the TIPI score is not merely an inflammation index but rather a “biological balance index,” differentiating it from SII-based models such as that proposed by Zhang et al. [28].

Recent studies have also highlighted that response to neoadjuvant therapy in breast cancer may be influenced not only by intrinsic tumor characteristics but also by host-related biological factors. For example, pretreatment blood selenium level has recently been associated with pathological complete response in patients with HER2-positive and triple-negative breast cancer receiving neoadjuvant therapy, supporting the relevance of systemic host milieu in treatment sensitivity [29]. In a large HER2-positive cohort, inflammatory blood markers such as monocyte-to-lymphocyte ratio (MLR) and neutrophil-to-lymphocyte ratio (NLR) showed regimen-dependent associations with pCR, particularly among patients receiving HER2-targeted therapy [22]. In addition, pretreatment systemic inflammation indices such as systemic inflammation response index (SIRI) have been associated with pCR and longer-term outcomes in HER2-positive breast cancer [23]. In parallel, emerging translational data suggest that resistance to HER2-targeted therapies may be driven by diverse molecular mechanisms beyond conventional clinicopathological variables [30]. Taken together, these findings support the broader conceptual rationale for incorporating systemic host inflammatory status into response prediction, while also highlighting that individual immune-inflammatory markers may have context-dependent performance and are unlikely to be sufficient in isolation. Within this context, the conceptual value of the TIPI score lies in integrating tumor proliferation, local immune infiltration, histological aggressiveness, and systemic inflammatory burden into a single biologically informed and clinically accessible composite index.

Within this broader biological context, hormone receptor (HR) negativity emerged as the most consistent factor associated with pCR in our adjusted analysis. This finding is in line with previous evidence indicating that HR-positive/HER2-positive tumors may show reduced sensitivity to anti-HER2-based neoadjuvant therapy because of the biological crosstalk between the HR and HER2 signaling pathways [1,31]. By integrating local proliferative activity, immune infiltration, histological aggressiveness, and systemic inflammatory burden, the TIPI score may partly reflect this broader biological complexity. However, the adjusted association of TIPI with pCR should be interpreted cautiously given the wide confidence interval and limited precision of the multivariable estimates in this relatively small cohort.

The added value of the TIPI score lies in its integrative and pragmatic design. Rather than relying on a single biological domain, TIPI combines tumor proliferation, histological aggressiveness, local immune infiltration, and systemic inflammatory burden within a unified pretreatment framework. In this respect, its novelty is conceptual as well as practical: it translates multidimensional tumor–host biology into a composite score derived from routinely available pathology reports and complete blood count parameters. Therefore, TIPI should not be viewed as a replacement for genomic assays, but rather as an accessible complementary tool for biological risk stratification in settings where advanced molecular testing is unavailable or selectively used. Accordingly, TIPI should be regarded as an exploratory composite score reflecting a simplified integration of selected tumor- and host-related variables rather than a comprehensive representation of the biological complexity underlying treatment response in HER2-positive breast cancer.

From a clinical implementation perspective, the TIPI score may be best positioned as a pretreatment adjunctive risk-stratification tool rather than a standalone decision-making instrument. Because it can be calculated from routinely available core-biopsy pathology data and baseline complete blood count parameters, it may provide a practical and low-cost biological layer to complement standard clinicopathological assessment, including clinical stage and hormone receptor status. In this context, a higher TIPI score may help identify patients with a biologically favorable profile who could be considered for future de-escalation strategies within prospective trials, whereas a lower TIPI score may support the need for closer monitoring, additional biological risk refinement, or consideration of escalation-oriented treatment frameworks. Importantly, the potential value of TIPI lies in complementing rather than replacing existing biomarkers. Future multimodal models may integrate TIPI with genomic assays, circulating tumor deoxyribonucleic acid (DNA) dynamics, and artificial intelligence (AI)-assisted digital pathology to improve predictive precision. However, given the modest discriminative performance of the score and the lack of external validation, these applications should currently be regarded as exploratory rather than practice-changing.

Although the biological components of the TIPI score may also be relevant in other breast cancer subtypes, the current model was developed and evaluated exclusively in HER2-positive disease treated with neoadjuvant chemotherapy plus anti-HER2 therapy. Therefore, its predictive relevance is currently supported only within this specific biological and therapeutic context. The present TIPI formula and cut-off should not be directly extrapolated to other breast cancer subtypes, such as hormone receptor-positive/HER2-negative or triple-negative disease, without dedicated subtype-specific derivation and external validation.

A major limitation of the present study is that the TIPI score was developed and evaluated in the same single-center retrospective cohort without external validation. Therefore, the current findings should be considered exploratory and hypothesis-generating rather than definitive evidence of generalizable predictive performance. Although we considered the possibility of validating the score in publicly available datasets, direct validation was not feasible because the simultaneous availability of pretreatment Ki-67, stromal TILs, histological grade, and complete blood count-derived parameters required for SII calculation was insufficient for direct score reconstruction. Accordingly, independent validation in larger, preferably multicenter, cohorts is required before the TIPI score can be considered for routine clinical application.

In addition, the multivariable estimates in the present study were characterized by wide confidence intervals, indicating limited precision and possible instability related to the relatively small sample size. Given the limited sample size and the derivation of both the scoring system and the multivariable model within the same cohort, a risk of model overfitting cannot be excluded. These factors may have affected the reliability and reproducibility of the adjusted estimates; therefore, the multivariable findings should be interpreted cautiously until confirmed in larger independent datasets.

Future studies should investigate the predictive performance of the TIPI score in treatment regimens incorporating next-generation antibody-drug conjugates (ADCs), such as trastuzumab deruxtecan (T-DXd) [32], and evaluate its correlation with emerging liquid biopsy parameters, including circulating tumor DNA (ctDNA) clearance [33]. Furthermore, the multimodal framework of the TIPI score could be further refined through integration with AI-based digital pathology platforms for TIL assessment, potentially enhancing its predictive precision in the era of personalized oncology.

## 5. Conclusions

In conclusion, the TIPI score was developed as a preliminary composite model combining tumor proliferation, local immune infiltration, histological aggressiveness, and systemic inflammatory status in the neoadjuvant setting of HER2-positive breast cancer. In this single-center retrospective cohort, the score showed an exploratory association with pathological complete response. However, given the modest discriminative performance, limited precision of the adjusted estimates, and lack of external validation, these findings should be interpreted cautiously. Further validation in larger independent, preferably multicenter, cohorts is required before the TIPI score can be considered a generalizable predictive tool or applied in routine clinical practice.

## Figures and Tables

**Table 1:** Baseline Clinicopathological Characteristics of the Patient Cohort and Their Association with Pathological Complete Response (pCR).

Variable	Total Cohort (n = 75)	pCR (+) (n = 34)	pCR (−) (n = 41)	*p*-Value
**Age (Years), Mean ± SD**	46.6 ± 11.1	45.4 ± 10.1	47.7 ± 11.9	0.380
**Menopausal Status, n (%)**				0.980
Premenopausal	44 (58.7)	20 (45.5)	24 (54.5)	
Postmenopausal	31 (41.3)	14 (45.2)	17 (54.8)	
**Clinical T Stage, n (%)**				0.085
cT1	26 (34.7)	7 (26.9)	19 (73.1)	
cT2	37 (49.3)	22 (59.5)	15 (40.5)	
cT3–cT4	12 (16.0)	5 (41.7)	7 (58.3)	
**Clinical N Stage, n (%)**				0.753
cN0	17 (22.7)	9 (52.9)	8 (47.1)	
cN1	43 (57.3)	18 (41.9)	25 (58.1)	
cN2–cN3	15 (20.0)	7 (46.7)	8 (53.3)	
**Clinical Stage Group, n (%)**				0.840
Stage IIA	38 (50.7)	16 (42.1)	22 (57.9)	
Stage IIB	14 (18.7)	8 (57.1)	6 (42.9)	
Stage IIIA–C	23 (30.7)	10 (43.5)	13 (56.5)	
**Hormone Receptor Status**				**0.011** *
Negative	26 (34.7)	17 (65.4)	9 (34.6)	
Positive	49 (65.3)	17 (34.7)	32 (65.3)	
**HER2 Expression (IHC/FISH)**				**0.028** *
IHC 3+	60 (80.0)	31 (51.7)	29 (48.3)	
IHC 2+/FISH (+)	15 (20.0)	3 (20.0)	12 (80.0)	
**Histological Grade**				**0.020** *
Grade 2	33 (44.0)	10 (30.3)	23 (69.7)	
Grade 3	42 (56.0)	24 (57.1)	18 (42.9)	
Ki-67 Proliferation Index **				0.093
≤20%	6 (8.0)	1 (16.7)	5 (83.3)	
>20%	69 (92.0)	33 (47.8)	36 (52.2)	
**Stromal TILs Category**				**0.012** *
Low (<10%)	32 (42.7)	8 (25.0)	24 (75.0)	
Intermediate (10–40%)	25 (33.3)	14 (56.0)	11 (44.0)	
High (>40%)	18 (24.0)	12 (66.7)	6 (33.3)	
**TIPI Risk Group**				**0.011** *
Low (≤11.41)	27 (36.0)	7 (25.9)	20 (74.1)	
High (>11.41)	48 (64.0)	27 (56.3)	21 (43.8)	

Abbreviations: pCR, pathological complete response; SD, standard deviation; IHC, immunohistochemistry; FISH, fluorescence in situ hybridization; TILs, tumor-infiltrating lymphocytes; TIPI, Tumor-Immune-Proliferation-Inflammation. * Indicates statistical significance (*p* < 0.05). ** Ki-67 categorization was used for descriptive baseline comparison only.

**Table 2:** Multivariable Logistic Regression Analysis of Factors Predicting Pathological Complete Response (pCR).

Variable	B	S.E.	Wald	OR (95% CI)	*p*-Value
**High TIPI Score** **(>11.41)**	0.888	0.572	2.406	**2.427 (0.791–7.462)**	**0.121**
**HR Negativity**	1.326	0.562	5.569	**3.765 (1.252–11.321)**	**0.018**
**Histological Grade** **(G3 vs. G2)**	1.038	0.577	3.238	**2.825 (0.912–8.772)**	**0.072**
Clinical Stage	-	-	1.221	-	0.875

Abbreviations: B, regression coefficient; CI, confidence interval; G, histological grade; HR, hormone receptor; OR, odds ratio; pCR, pathological complete response; S.E., standard error; TIPI, Tumor-Immune-Proliferation-Inflammation. Indicates statistical significance (*p* < 0.05). Model fit was assessed using the Omnibus test (*p* = 0.025) and Nagelkerke R^2^ (0.256).

## Data Availability

The datasets generated and analyzed during the current study are available from the corresponding author on reasonable request due to institutional and privacy regulations.
